# Personalized breathing recommendations for anxiety relief: a data-driven exploratory approach focusing on feasibility

**DOI:** 10.7717/peerj.21483

**Published:** 2026-08-04

**Authors:** Dongyin Zhuo

**Affiliations:** Munich, Bavaria, Germany

**Keywords:** Deep learning, Personalization, Anxiety reduction

## Abstract

Breathing exercises are widely used to alleviate anxiety, but most interventions use fixed templates of inhalation, exhalation, and repetition. This study preliminarily explores the feasibility of data-driven personalization of breathing recommendations for general individuals. We hypothesized that based on the characteristics of individual users, a data-driven method could give a feasible breathing intervention suggestion without the need of specialized hardware. A multilayer perceptron was trained on questionnaire responses to predict the inhalation, exhalation, and repetition times. The model stability was assessed using several independently trained models and exhibited high consistency with coefficients of variation below 10% across all outputs. A single-blind randomized study (*N* = 820), which compared the personalized recommendations to a fixed template that is known to effectively reduce anxiety, measured the outcomes in terms of anxiety reduction and user-reported satisfaction. The main analysis and subgroup analysis both detected no statistically significant differences in effectiveness between model recommendation and fixed template after applying multiple comparison corrections. However, adults aged 18–30 years with the medium anxiety level showed a moderate exploratory preference for the fixed template. These findings indicate that individual characteristics may influence the preference of breathing interventions. Overall, the results highlight the feasibility of datadriven personalization in non-medical anxiety interventions. The outcomes can still be enhanced by various methods such as collecting more training data, fine-tuning on the model, or choosing advanced machine learning or offline reinforcement learning approaches.

## Introduction

Generalized Anxiety Disorder (GAD) is a prevalent psychiatric condition linked to decreased patient productivity and substantial somatic symptoms (Wittchen, 2002; Davidson et al., 2010). Taking the mental harm of GAD into account, finding the reason causing GAD and designing targeted prevention against GAD for the general population is essential. The research of Rowa et al. (2017) discovered that chronic anxiety and stress are key risk factors for its development. Therefore, mitigating daily anxiety plays a critical role in GAD prevention. Developing easily accessible intervention solutions for the general population to help them better manage their daily anxiety level is crucial for GAD prevention and public mental-health improvement.

Several non-medical treatments such as mindfulness and deep breathing exercises have demonstrated efficacy in managing GAD (Smith et al., 2015; Steffen et al., 2021). In particular, deep breathing has been shown to enhance the Heart Rate Variability (HRV), which is a physiological marker associated with anxiety levels (Chalmers et al., 2014). However, the reliable measurement of HRV presents significant practical challenges. The biggest challenge is the measurement accuracy of HRV. The most common way of calculating HRV is based on Electrocardiogram, therefore wearable devices are required for the measuring, which reduces its accessibility (Li et al., 2023). Even though Alam et al. (2020) developed a HRV measurement method based solely on face video, the accuracy of this method is still under discussion. Furthermore, the HRV is susceptible to external factors such as smoking and caffeine intake (Karakaya et al., 2007; Zimmermann-Viehoff et al., 2016). Additionally, sustained adherence to deep breathing techniques is challenging (Steffen et al., 2021). To address these limitations, we proposed a personalized approach whose measurement of anxiety level is based on validated self-reported questionnaires as an easier accessible solution. It enables the general population to receive interventions in daily life without special hardware.

Most existing breathing exercises follow fixed templates. This approach offers little flexibility because they give the same inhalation time, exhalation time, and repetition to everyone. To address these issues, we propose an adaptive breathing guidance system that tailors intervention parameters for user to relieve anxiety by deep breathing intervention. Breitborde et al. (2015) investigated personalized breathing intervention based on the breathing frequency, but this approach overlooks the distinct inhalation and exhalation durations of individuals. On this basis, the study by Vlemincx & Cortez-Vázquez (2024) concluded that such methods, which focus solely on breathing frequency while neglecting the balance of inhalation and exhalation durations, are not suitable for all individuals. The study also noted that different inhalation-exhalation ratio showed various effects for different individuals, which provides a theoretical basis for personalization. Therefore, we recommend an individualized adjustment of both phases to enable more precise, effective personalization.

The system was implemented using Machine Learning and trained on user-provided data, which were collected through questionnaires to describe the personalized settings and evaluations for deep breathing exercises. A custom loss function was designed to guide the model training by dynamically adjusting its penalties according to the prediction effectiveness, user satisfaction, and established theoretical foundations. To facilitate accurate practice, we created a website that used the trained model to generate animations synchronized with specified breathing rhythms (JustBreath, 2025a; JustBreath, 2025b).

Unlike prior studies, our approach integrates user-profile-based personalization with breathing exercises. Recognizing the limitations of physiological indicators in capturing the emotional states, we incorporated subjective assessments to enhance the emotional relevance. We hypothesized that personalized breathing methods would reduce anxiety in a comparable manner to fixed templates while offering higher user satisfaction.

The remainder of this article is organized as follows: ‘Methods’ details the methods for intervention design, evaluation, and data analysis. ‘Results’ presents the experimental results. ‘Discussion’ discusses the main findings, implications, and limitations. ‘Conclusion’ concludes the paper.

## Methods

### Participant recruitment and ethics

Participants were recruited online *via* the Testable platform (Rezlescu et al., 2020). The eligibility conditions required that participants were at least 18 years old, legally capable of providing consent, and self-reporting in good health.

No personally identifiable information (PII) was collected during the study. All data were stored on the Testable platform using European General Data Protection Regulation (GDPR)-compliant servers, which were hosted by Hetzner GmbH.

To ensure accurate delivery of the intervention to the participants, we developed a website that featured animated demonstrations of the breathing process. The website collected no user data, and its identical version was deployed *via* GitHub (JustBreath, 2025a; JustBreath, 2025b).

The studies involving human participants were reviewed and approved by The Ethical Committee of Nankai University Affiliated Tianjin Fourth Central Hospital (Ethical NO. SZXLL-2024-018). Informed consent was obtained *via* a mandatory electronic checkbox that participants had to select before proceeding to the experimental tasks.

### Questionnaire design

#### Data collection for training

Williams, Morlock & Feltner (2010) demonstrated that the Global Anxiety-Visual Analog Scale (GA-VAS) effectively detected short-time changes in anxiety levels based on patient-reported data. Therefore, we used this scale to assess the anxiety levels of the participants before and after the intervention and their satisfaction with its duration. Accordingly, the GA-VAS then served both as an input term of training data and as outcome measure for evaluation of anxiety reduction. A Likert scale was used to capture the expectations of the participants regarding the intervention intensity. The overall satisfaction was quantified using a variant of the Client Satisfaction Questionnaire (CSQ), the CSQ-4 (Boß et al., 2016; Pedersen et al., 2023).

The GAD-2 scale was initially included in the questionnaire to assess participants’ anxiety levels. However, after data collection began, we recognized that GAD-2 measures trait anxiety over the past two weeks, which does not adequately capture the state anxiety changes targeted by our intervention. Consequently, we made a *post hoc* decision that GAD-2 responses were omitted from the final analysis, in order to better focus on the state anxiety level (Byrd-Bredbenner, Eck & Quick, 2021). Nevertheless, sensitivity analysis of GAD-2 was conducted to preliminarily explore the influence of trait anxiety on our proposed approach (see Appendix A).

As the GA-VAS is validated to capture changes in anxiety levels before and after the intervention, we used the GA-VAS results before receiving the intervention as an indicator of anxiety assessment in this phase. As the generation of the personalized intervention relied on GA-VAS results during the intervention, this adjustment did not affect the accuracy of the experimental process.

On our website, participants could adjust the inhalation and exhalation durations and set repetition counts (JustBreath, 2025a) as guided by the animations. Kreibig (2010)’s study showed that longer exhalation time reduced anxiety levels and improved the HRV, so we added a non-mandatory advisory.

#### Data collection for evaluation

The questionnaire closely mirrored the structure described in ‘Data Collection for Training’. Consequently, while designing this questionnaire, we decided not to use the GAD-2 scale for data collection to better evaluate the state anxiety level and maintain the methodological consistency across all experimental stages. To more accurately assess the pre-intervention anxiety, we supplemented the GA-VAS with the State Anxiety (S-Anxiety) component of the State-Trait Anxiety Inventory (STAI) scale (Spielberger et al., 1971; Spielberger et al., 2015). Participant satisfaction with the intervention was re-evaluated using the CSQ-4.

Our developed website had a single-blind design for the intervention. Participants were asked to provide their expected intensity, intervention duration, age group (optional) and gender (optional). A pseudo-random number generator (PRNG) on the server side determined whether the participants received a fixed template or a recommendation from a deep learning model. The PRNG’s statistical uniformity ensured balanced allocation between conditions over time (L’Ecuyer, 2010). Although participants were assigned a group identifier for data tracking, they remained blinded to the mapping between these identifiers and the specific intervention strategies (fixed *vs.* personalized). Steffen et al. (2021) found that a 10-second deep breath optimally improved the HRV, so we selected this duration as the template of the control group. In the referenced study, the specific inhalation-to-exhalation ratio was not reported. Therefore, for the control group in our study, we adopted an equal division of inhalation and exhalation durations as a standardized and neutral baseline in the absence of more detailed guidance. However, we acknowledge that our utilization of this intervention may not be the optimal solution, which potentially limits the comparative contrast of our experiment. The intervention duration for the control group approximated the mean of the shortest and longest durations that the model predicted. Pseudo-random numbers were generated using the secrets module of Python (version 3.9).

Participants were assigned a group identifier for the intervention (*e.g.*, Spade or Club) but were unaware of its association with the model (JustBreath, 2025b). To filter out invalid responses, we incorporated attention tests. Participants were asked to report the group identifier of the intervention that they were assigned during the intervention in the questionnaire, and invalid distractor options (*e.g.*, Heart and Diamond) were included among the choices. In CSQ-4, we added a question to prompt the participants to select “does not apply to me” if they had carefully completed the test. For responses where participants failed the attention tests, a score of -1 was assigned to the CSQ-4 scale regardless of their other inputs.

### Model implementation

#### Data pre-processing

To ensure the quality of the training set, we filtered and processed the collected data.

We calculated the difference between the mean CSQ-4 score and the GA-VAS values before and after the intervention. For training the model with data where people felt satisfied and got their anxiety relieved, we made a design decision that only questionnaires with a mean CSQ-4 score above 3 (*i.e.,* no worse than “does partly apply to me” for each question) and a lower GA-VAS score post-intervention were used for model training.

For participants who felt that the intervention duration was too short or too long, we adjusted the set duration as follows:

(1) \begin{eqnarray*}{t}_{{i}_{new}}={t}_{i}(1-\sin \nolimits ( \frac{s}{100} ))\end{eqnarray*}

where *t*_*i*_*new*__ is the adjusted inhalation, exhalation, or repetition time; *t*_*i*_ is the corresponding original participant-set value; *s* is the satisfaction with the intervention duration, scaled from –50 (“too short”) to 50 (“too long”). The adjusted inhalation and exhalation time lengths were rounded to the nearest millisecond, and the number of repetitions was rounded to the nearest integer.

We chose the sin function to make this adjustment because of its non-linear sensitivity. Within the definition domain of [ − 1, 1], the function has its maximum derivative at zero and minimum at the edge of boundary, which means it will demonstrate a dampening effect that prevents over-adjustment at extreme inputs.

As of April 11th, 2025, 700 questionnaire responses were collected. After applying the filtering and adjusting criteria, 402 valid responses were used as the training set for the model.

#### Model architecture

**Figure 1:**
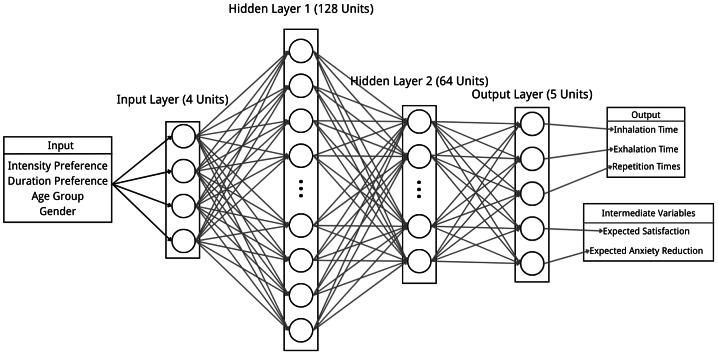
Model architecture as a multilayer perceptron for personalized breathing intervention recommendations.

The deep learning model was implemented as a multilayer perceptron (MLP) with two hidden layers. The model’s input included four user-specific variables: expected intensity, expected intervention duration, age group, and gender. The output comprised five variables: inhalation time, exhalation time, number of repetitions, expected anxiety reduction, and expected satisfaction. Figure 1 illustrates the model structure.

A robust scaler was used to normalize all inputs and de-normalize the outputs to mitigate the effects of outliers, prevent gradient vanishing, avoid overfitting during training, and ensure numerical stability (de Amorim, Cavalcanti & Cruz, 2023; Ahsan et al., 2021; Ven & Lederer, 2021).

The two hidden layers of the model contained 128 and 64 neurons. Both hidden and output layers used the tanh activation function to handle both positive and negative input values due to regularization. To mitigate gradient vanishing caused by the activation function in deeper networks, we used a shallow architecture (Glorot & Bengio, 2010).

The model was trained using the Adam optimizer with a default learning rate of 0.001. A custom loss function was developed to optimize both prediction accuracy and output plausibility. The Mean Absolute Error (MAE) served as the primary evaluation metric during training. The model was trained for 500 epochs. Although the training loss had not fully plateaued, further training increased the risk of overfitting due to the small size of the model and structured nature of the task. The 500-epoch limit was selected to balance convergence and generalization. The network contained 9,221 trainable parameters.

The deep learning model was implemented using TensorFlow (version 2.19.0) with the Keras API (version 3.9.1), where custom loss functions and training procedures were defined (Abadi et al., 2015; Chollet et al., 2015). Data processing, including model training and data analysis, was performed using Numpy (version 2.0.2) and Pandas(version 2.2.3) (Harris et al., 2020; McKinney, 2010). Joblib (version 1.4.2) was used to locally store the scalers (Joblib, 2020).

#### Design of custom loss

We implemented a custom loss function (‘Model Architecture’) to align the model outputs with the requirements in ‘Data Pre-processing’. The loss function is defined as follows:

(2) \begin{eqnarray*}L(T)=MSE(T)+{\lambda }_{1}S(5(3-{t}_{s}))+{\lambda }_{2}S(-5{t}_{r})+{\lambda }_{3}S(5({t}_{i}-{t}_{e}))\end{eqnarray*}

where *L*(*T*) is the total loss for a single prediction; *T* is the vector of predicted outputs *t*_*n*_: *t*_*i*_ for the inhalation duration, *t*_*e*_ for the exhalation duration, *t*_*r*_ for the predicted anxiety reduction, and *t*_*s*_ for the predicted satisfaction). The sigmoid function is defined as $S(x)= \frac{1}{1+{e}^{-x}} $. The penalty weights were set to *λ*_1_ = *λ*_2_ = 0.25, and *λ*_3_ = 0.5.

When the model’s output indicated that the average satisfaction was below 3, the predicted anxiety reduction was negative (*i.e.,* anxiety did not decrease or actually increased), or the inhalation duration exceeded the exhalation duration, additional penalties were applied to the loss function. The satisfaction and anxiety reduction were consistent with the findings in ‘Data Pre-processing’. The requirement for the inhalation and exhalation durations is based on the findings of Kreibig (2010) where a higher exhalation-to-inhalation duration ratio could improve the HRV of the participants.

To ensure smoother gradients during backpropagation and prevent abrupt shifts in the loss surface, the sigmoid function was used instead of a step-wise penalty. This approach enhanced stable convergence while enforcing meaningful regularization effects.

### Evaluation strategy

#### Evaluation of the model stability

To assess the reliability and consistency of the outputs of the deep learning model, a stability analysis was conducted using structured input probing. A synthetic test set of 50 samples was generated, where input features were systematically varied. In each subgroup of 10 samples, only the expected intensity of intervention was linearly increased in increments of 10, while all other variables remained constant. This setup enabled controlled comparisons across defined input conditions.

An ensemble of 10 independently trained models, which only differed in random seed initialization and data shuffling, was used to predict the outputs for this test set. For each test sample and each output variable, we computed the mean coefficients of variation (CV) and mean maximum differences (mean-max diff) across the predictions from the 10 models. These metrics were used to quantify the inter-model variability and evaluate the stability of the model outputs under minor training perturbations. Lower variance among the models confirmed higher output consistency and consequently greater confidence in the learned patterns of the model.

#### Participant-based evaluation of the model

**Figure 2:**
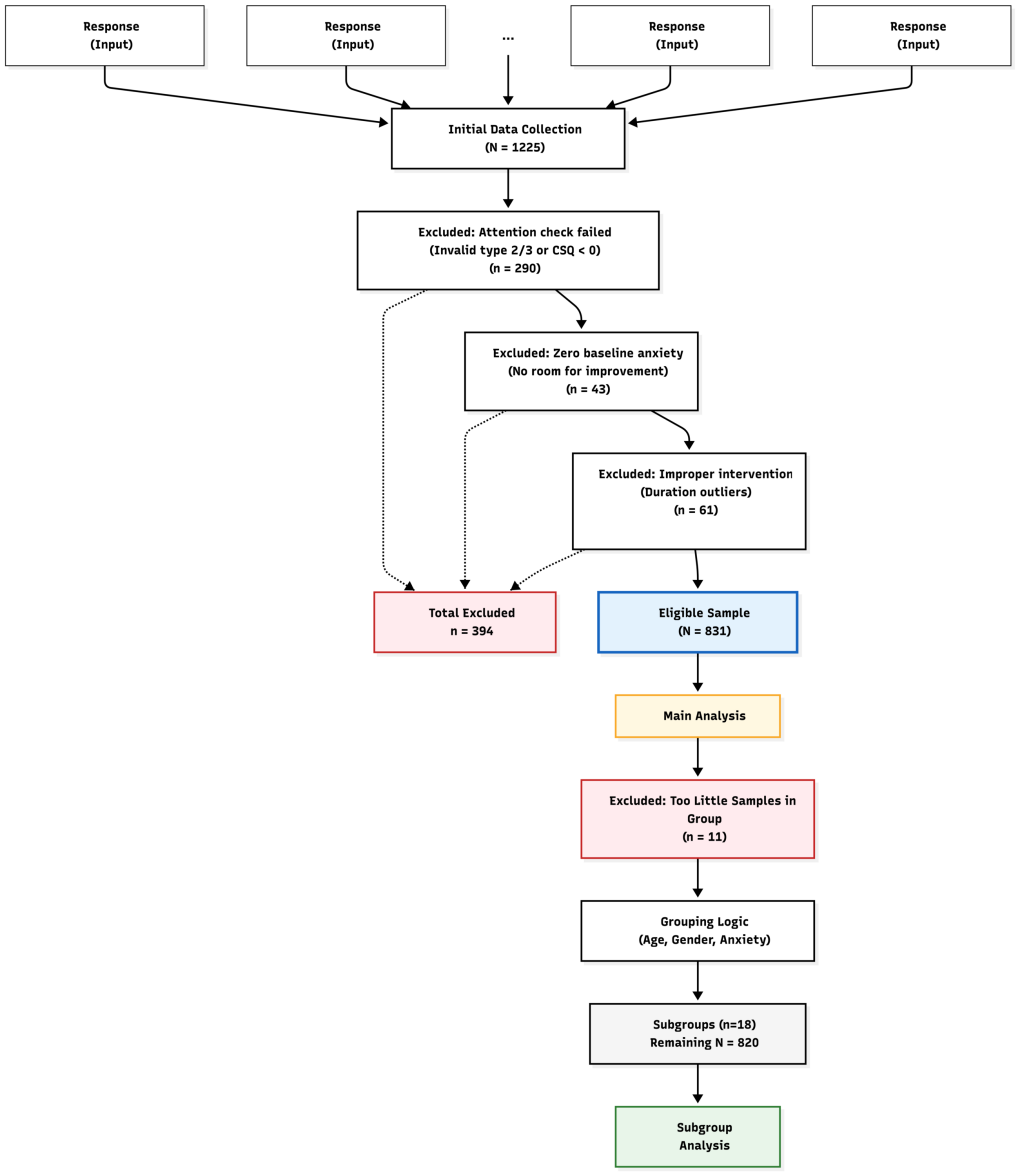
Flowchart of the participant enrollment, data filtering, and sub-grouping process for analysis.

To ensure the reliability of the analysis outcomes, we applied a systematic data pre-processing and sub-grouping strategy. Figure 2 shows the flowchart of this process.

 Since some participants may have provided arbitrary or inattentive responses, data filtering was performed to ensure data quality. Responses that failed the attention tests referred to in ‘Data Collection for Evaluation’ were excluded. In addition, questionnaires that were completed in less time than the minimum duration of a single intervention (44.218 s and 60 s for the model recommendation and fixed template groups, respectively) were removed. The minimal intervention duration of model recommendation was the minimal intervention time among all possible inputs.

Moreover, certain data points were flagged as “problematic” if the GA-VAS scale value pre-intervention was set to 0, which indicates that the participant reported no anxiety and could not exhibit a reduction in anxiety, or if the response time deviated from the normal response time range, which is expressed as follows:

(3) \begin{eqnarray*}max({Q}_{1}-1.5IQR,{t}_{min})\leq t\leq {Q}_{3}+1.5IQR\end{eqnarray*}

where *Q*_1_ and *Q*_3_ are the first and third quartiles, respectively; *IQR* is the interquartile range of all data of questionnaire completion time; *t* is the questionnaire completion time of a single participant. The marked data were not used in the main analysis. The exact data filtering details are also included in Fig. 2.

This analysis focuses on the CSQ questionnaire scores of the participants and the difference (reduction) in GA-VAS values measured before and after the intervention. To reflect the relative intensity of the intervention, we introduced the following reduction percentage:

(4) \begin{eqnarray*}{p}_{r}= \frac{{g}_{pre}-{g}_{post}}{{g}_{pre}} \end{eqnarray*}

where *p*_*r*_ is the reduction percentage, *g*_*pre*_ is the GA-VAS score before the intervention, and *g*_*post*_ is the GA-VAS score after the intervention. Since *g*_*pre*_ can be 0, calculations involving it may yield NaN and −Inf. After data cleansing, the main dataset no longer contained data that could cause this situation. However, if all data “without factual errors” were analyzed, this issue would hinder further analysis. To avoid interference, data with these characteristics were excluded from the calculation of this indicator.

First, we selected Shapiro–Wilk test to assess the normality of these variables (Shapiro & Wilk, 1965). However, the work of Kim (2013) suggests that this method may be unreliable for large sample sizes (*N* > 300), so we introduced the skewness and kurtosis as auxiliary criteria.

The analysis (Fig. 2 and ‘Normality Test’) reveals that the variables were not normally distributed. Consequently, we used Brunner–Munzel test to compare the model recommendation and fixed template groups (Brunner & Munzel, 2000). For several subgroups, because their sample sizes are too small (model *N* < 3, template *N* < 3), the Brunner–Munzel test could not be executed. We therefore excluded these groups and their corresponding samples (*N* = 11). The Cliff’s delta was applied to measure the effect size along with its 95% Confidence Interval (CI) for better interpretation on effect size (Cliff, 1993). We used Bootstrapping to calculate the CI, since the study of Justus, Rodrigues & Dos Santos Sousa (2024) discovered that this method demonstrates high robustness for making it.

To avoid the multiple testing problem inherent in conducting subgroup analyses, *p*-values were adjusted using the Benjamini–Hochberg False Discovery Rate (FDR) correction, in order to provide FDR-adjusted *p*-values (*q*-values), which enhanced the statistical accuracy by limiting false significance (Benjamini & Hochberg, 1995).

For the subgroup analysis, we used the anxiety level before intervention, age group, and gender as the grouping variables. Principal Component Analysis (PCA) was used to integrate the GA-VAS and STAI anxiety measures and to improve the classification accuracy. The first principal component was extracted and used to group the data into three groups. Subsequently, the data were subdivided by age and gender. To avoid too many subgroups with a low sample size, we merged the age groups into: 18–30 years old, 31–50 years old, 51+ years old, and unspecified. Gender was categorized as male, female, and unspecified. Theoretically, there were 36 subgroups, but only those with at least 10 participants per condition (model recommendation and fixed template) were included in the statistical analysis, and smaller subgroups were excluded.

All analyses were conducted using Python and the Scipy (version 1.13.1) and Scikit-learn (version 1.6.1) libraries (Virtanen et al., 2020; Pedregosa et al., 2011).

## Results

### Model stability analysis

To evaluate the stability of machine learning models, we analyzed the inter-model variance across 12,120 synthetic test samples. The standard deviation and maximum prediction difference for each sample and output dimension were computed.

Overall, the models exhibited low inter-model variance, which indicates consistent predictions across random initializations for key output variables (inhalation time, exhalation time, and repetition times), as reflected by their mean coefficients of variation (CV) and mean maximum differences (mean-max diff). Other output variables, which were only used to determine the loss function (‘Design of Custom Loss’), were excluded from the inter-model variance analysis.

**Figure 3:**
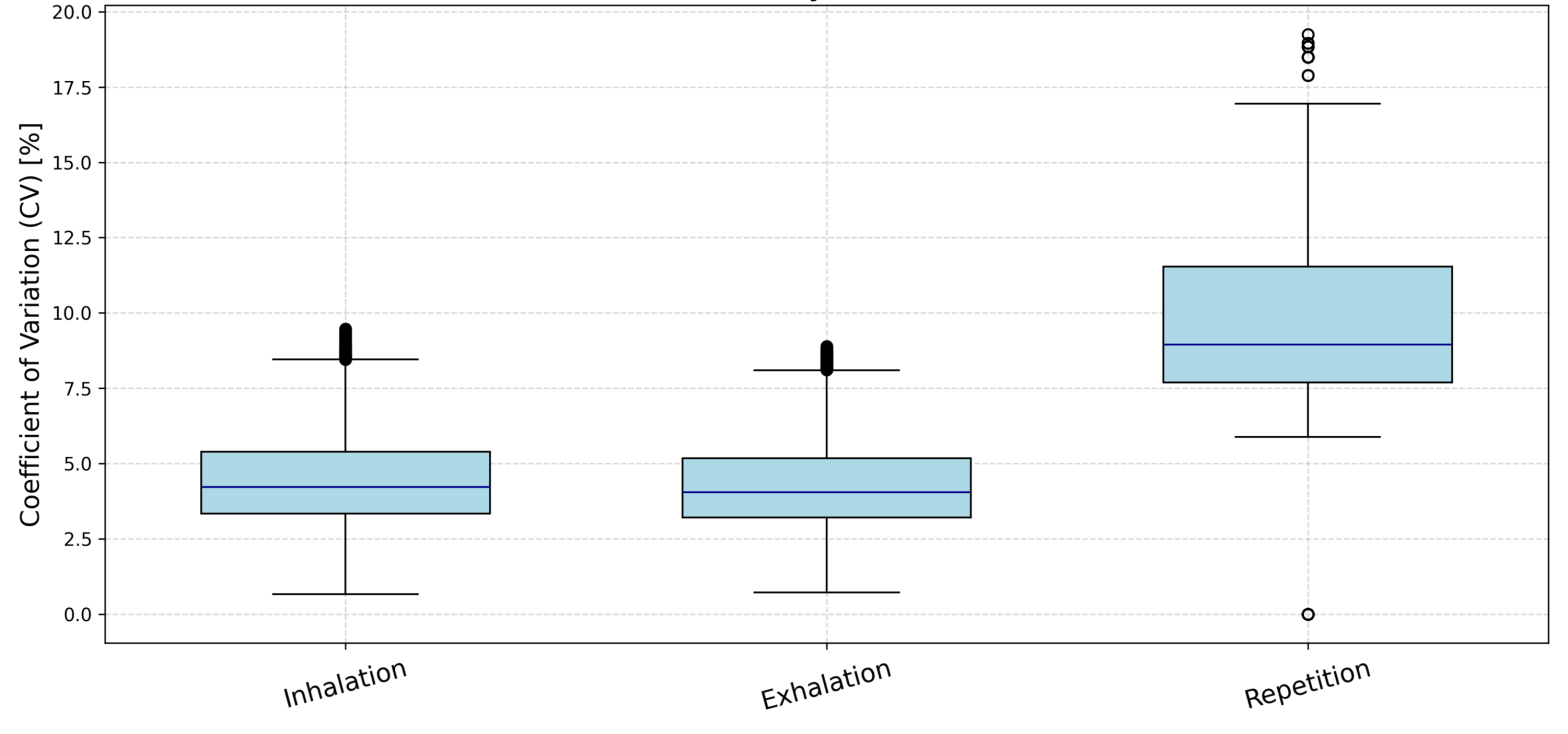
Boxplots of the CV across test samples for inhalation, exhalation, and repetition time lengths. The boxes denote the interquartile range (IQR), the horizontal lines signify the medians, and the whiskers extend to 1.5 × IQR.

The mean CV values for the inhalation, exhalation, and repetition time lengths were 4.43%, 4.24%, and 9.41%, respectively (Table 1). Figure 3 illustrates a consistently low CV distribution across all outputs.

**Table 1:** Mean CV values for each primary output across models. Lower CV values indicate higher model output stability.

Variable	Mean CV (%)
Inhalation	4.43
Exhalation	4.24
Repetition	9.41

No universal threshold defines the acceptable coefficient of variation (CV). Previous studies reported 7.5%–20% depending on the measurement domain (Shechtman, 2013). In this study, CVs below 10% for the inhalation, exhalation, and repetition outputs suggest high measurement consistency across models. Since the number of repetitions is a discrete quantity, continuous model predictions were rounded to integers during post-processing, which made minor fluctuations insignificant on the final discrete output. Therefore, the inter-model variance of this variable was also considered stable.

### Participant-based evaluation results

#### Data collection

As of 26th March 2026, 1,225 questionnaire responses were collected. After data cleaning, 831 responses were retained for the main analysis. 417 of them were assigned to the personalized group, and 414 of them were assigned to the fixed template group.

#### Normality test

Table 2 summarizes the normality test results. The Shapiro–Wilk test rejected the normality assumption for all variables (*P* < 0.001). Skewness was high for the CSQ variable in the model-generated recommendation group and reduction percentage in the fixed-template group (|*skewness* | ≥ 1), which indicates a deviation from normality.

Considering the distributional differences of each variable between the model-generated recommendation group and the fixed template group (|*Kurtosis*_*ai*_ − *Kurtosis*_*template*_| > 1 for all variables), the samples were not identically distributed. This finding violates the hypothesis of the Mann–Whitney U test, because this test assumes that the two groups of data have similar distribution patterns. The different shapes of the distributions of the two data sets can cause the Mann–Whitney U Test to be more likely to give false significance conclusions, even though the difference between the two data sets is not actually significant (Nachar, 2008).

#### Main analysis

Table 3 summarizes the main analysis results. No statistically significant differences were observed (*P* > 0.05) across the three variables. Consistent with the thresholds that Romano et al. (2006) proposed, the effect sizes were also minimal (|Cliff’s delta| < 0.147), which indicates no significant differences between these groups.

#### Exploratory subgroup analysis

**Figure 4:**
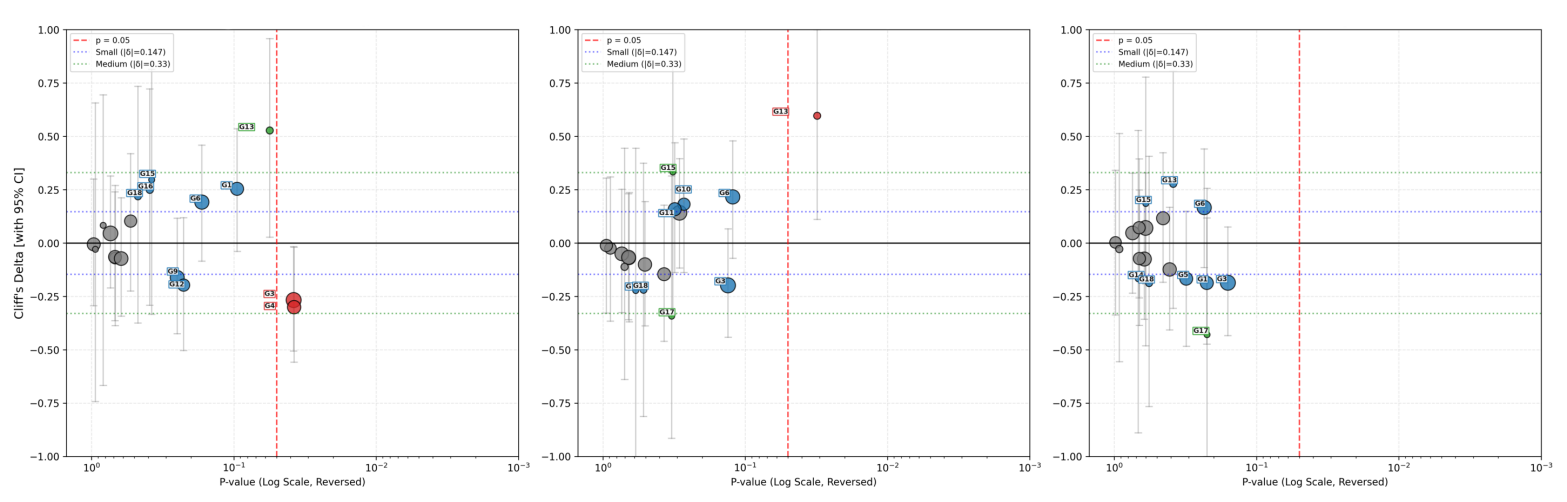
Scatter plots of the subgroup-level Cliff’s delta with 95% Confidence Interval and *P*-values across three outcomes: anxiety reduction, percentage reduction, and satisfaction. The points represent subgroups; Red: statistically significant (*P* < 0.05); Green: non-significant but medium effect size (—Cliff’s delta— ≥ 0.33); Blue: non-significant but detectable effect size (—Cliff’s delta— ≥ 0.147); gray: non-significant, negligible effect (—Cliff’s delta— < 0.147). The horizontal dashed lines indicate the thresholds for small (—Cliff’s delta— = 0.147) and medium (—Cliff’s delta— = 0.33) effect sizes. The vertical dashed line marks the conventional statistical significance threshold (*P* = 0.05).

To further explore the potential heterogeneity of our approach, subgroup analyses were conducted based on the age, pre-intervention anxiety levels, and gender of the participants. Table 4 shows the detailed results with a graphical description in Fig. 4. For Groups 13–17, where sample sizes were limited (model *N* < 10, template *N* < 10), the results and interpretations based on them are provided for illustrative purposes only.

Statistically significant differences between the model recommendation and the fixed template groups were only observed in several groups in the exploratory subgroup analysis. For the variable satisfaction, significant differences were found in Groups 3 (*q* = 0.038), and 4 (*q* = 0.038). The largest difference appeared in Group 13, which was the only subgroup with a medium effect size (|Cliff’s delta ≥ 0.33|); thus, there was an observable preference for the model-recommended breathing method. However, in Groups 3 and 4, this difference was not always positive, and there were significantly negative differences (Cliff’s delta < 0). This suggests that participants in these groups were more likely to find the fixed template more satisfying than the model’s recommendations.

**Table 2:** Shapiro–Wilk test result, skewness, and kurtosis for all analyzed variables. The reduction percentage range is [ − 99, 1], which reflects possible anxiety changes (decrease, no change, or increase) due to the changes in GA-VAS difference and contributes to the observed extreme kurtosis.

Variable	Group	Shapiro–Wilk *P*-value	Skewness	Kurtosis
Reduction	AI	5.7 × 10^−14^	1.125	2.566
Reduction	Template	7 × 10^−15^	0.667	3.646
CSQ	AI	4.3 × 10^−15^	−1.023	1.065
CSQ	Template	1.2 × 10^−12^	−0.832	0.758
Reduction Percentage	AI	7.9 × 10^−8^	−0.366	0.673
Reduction Percentage	Template	4.4 × 10^−29^	−6.339	72.296

**Table 3:** Differences in all measured variables across all participants after data cleaning.

Variable	*P*-value	Cliff’s Delta [95% CI]
CSQ	0.662	−0.017 [−0.089, 0.064]
Reduction	0.679	−0.017 [−0.094, 0.066]
Reduction_percentage	0.948	0.003 [−0.080, 0.085]

**Table 4:** Subgroup analysis results.

Group number	Age group	Anxiety level	Gender	Model N	Template N	SP	SΔ [95% CI]	RP	RPΔ [95% CI]	R	RΔ [95% CI]
1	Young	Low	Male	28	30	0.095	0.255 [−0.039, 0.536]	0.655	−0.070 [−0.369, 0.237]	0.224	−0.187 [−0.474, 0.118]
2	Young	Low	Female	21	25	0.683	−0.070 [−0.387, 0.271]	0.888	−0.025 [−0.366, 0.310]	0.983	0.004 [−0.337, 0.341]
**3**	**Young**	**Medium**	**Male**	38	39	**0.038**	**−0.267 [−0.506, −0.017]**	0.132	−0.198 [−0.441, 0.066]	0.159	−0.186 [−0.434, 0.076]
**4**	**Young**	**Medium**	**Female**	32	29	**0.038**	**−0.300 [−0.558, −0.019]**	0.507	−0.100 [−0.388, 0.194]	0.409	−0.124 [−0.407, 0.168]
5	Young	High	Male	35	22	0.687	−0.065 [−0.364, 0.240]	0.373	−0.145 [−0.460, 0.178]	0.312	−0.166 [−0.483, 0.149]
6	Young	High	Female	32	37	0.168	0.193 [−0.084, 0.459]	0.122	0.216 [−0.071, 0.479]	0.234	0.167 [−0.115, 0.441]
7	Middle-aged	Low	Male	38	36	0.738	0.045 [−0.211, 0.315]	0.290	0.143 [−0.116, 0.395]	0.603	0.071 [−0.192, 0.329]
8	Middle-aged	Low	Female	32	32	0.619	−0.072 [−0.342, 0.212]	0.737	−0.050 [−0.325, 0.252]	0.746	0.048 [−0.234, 0.328]
9	Middle-aged	Medium	Male	30	37	0.251	−0.161 [−0.424, 0.117]	0.662	−0.067 [−0.359, 0.232]	0.616	−0.075 [−0.357, 0.215]
10	Middle-aged	Medium	Female	29	22	0.533	0.103 [−0.224, 0.419]	0.270	0.182 [−0.138, 0.487]	0.669	0.072 [−0.257, 0.395]
11	Middle-aged	High	Male	26	32	0.968	−0.006 [−0.293, 0.300]	0.313	0.159 [−0.138, 0.470]	0.456	0.117 [−0.184, 0.423]
12	Middle-aged	High	Female	25	26	0.226	−0.197 [−0.503, 0.118]	0.949	−0.011 [−0.328, 0.305]	0.667	−0.072 [−0.386, 0.249]
**13**	**Older**	**Low**	**Male**	8	9	0.056	0.528 [0.028, 0.958]	**0.031**	**0.597 [0.111, 1.000]**	0.386	0.278 [−0.306, 0.806]
14	Older	Low	Female	6	6	0.830	0.083 [−0.667, 0.694]	0.588	−0.222 [−1.000, 0.444]	0.679	−0.167 [−0.889, 0.528]
15	Older	Medium	Male	3	9	0.379	0.296 [−0.333, 0.815]	0.323	0.333 [−0.333, 0.852]	0.602	0.185 [−0.481, 0.778]
16	Older	Medium	Female	12	6	0.391	0.250 [−0.292, 0.722]	0.705	−0.111 [−0.639, 0.444]	0.923	−0.028 [−0.556, 0.514]
17	Older	High	Male	7	5	0.944	−0.029 [−0.743, 0.657]	0.329	−0.343 [−0.914, 0.314]	0.223	−0.429 [−1.000, 0.257]
18	Older	High	Female	8	8	0.473	0.219 [−0.375, 0.734]	0.520	−0.219 [−0.812, 0.375]	0.570	−0.188 [−0.766, 0.406]

**Notes.**

SQ Satisfaction *q*-value SΔ Satisfaction Cliff’s delta RQ Reduction Percentage *q*-value RPΔ Reduction Percentage Cliff’s delta R Reduction *q*-value RΔ Reduction Cliff’s delta

Age groups are: 18–30 (“Young”), 31–50 (“Middle-aged”), and 51+ (“Older”). Bold font indicates statistically significant differences.

Although the differences in anxiety reduction were only statistically significant in the Group 13 (*q* = 0.031), small effects (|Cliff’s delta| > 0.147) were observed in Groups 3, 6, 10, 11, 14, and 18, and medium effect size (|Cliff’s delta| ≥ 0.33) was observed in group 13, 15 and 17 for reduction percentage. The effect sizes were negative in Groups 3, 14, 17, and 18, which favored the fixed template, and positive in Groups 6, 10, 11, 13 and 15, which favored the model-generated recommendations. However, this effect was actually small and insignificant, implying that participants in these groups had weak preferences for the above groups.

For the absolute anxiety reduction, the differences of all groups were insignificant. For this variable, small effects (|Cliff’s delta| > 0.147) were observed in Groups 1, 3, 5, 6, 13, 14, 15, 18, and medium effect size (|Cliff’s delta| ≥ 0.33) was observed in group 17. The effect sizes were positive in Groups 6, 15, and 13, and were negative in groups 1, 3, 5, 14, 17, and 18.

However, the 95% CI of all groups and all variables overlaps the range where the effect size is minimal (|Cliff’s delta < 0.147|), therefore even though this finding is informative, but we should only give an exploratory conclusion about which intervention performed better in these groups.

## Discussion

### Stability and feasibility

To preserve blinding, all interventions were assigned neutral group identifiers with uniform naming conventions across conditions to minimize the risk of preference bias. The participants remained unaware of whether their breathing routine was model-generated or derived from a fixed template. Consequently, placebo effects such as perceived personalization were likely balanced across groups and unlikely to confound the main comparisons (Hróbjartsson et al., 2014). However, while model stability is used as an indicator of model performance, it does not ensure that the personalization approaches are optimal or statistically significantly better than other solutions.

Analyses of the full dataset (‘Main Analysis’) showed no statistically significant differences between model-recommended breathing methods and fixed templates, with negligible effect sizes observed for both anxiety reduction and user satisfaction.

Exploratory subgroup analyses (‘Exploratory Subgroup Analysis’) did not show any statistically significant difference in anxiety reduction between model recommendation and fixed template in any of the non-illustrative subgroups (*N* ≥ 10). However, a slight preference for fixed template over model recommendation was observed in the group of young participants with medium anxiety levels. For the other subgroups, the intervention effects between model recommendation and fixed template remained statistically indistinguishable with effect sizes falling within a small or even negligible interval (|Cliff’s delta| < 0.33). One possible reason for this result is that the fixed template is proven to be an ideal breathing intervention method (Steffen et al., 2021; Edmonds et al., 2009; Lehrer & Gevirtz, 2014). Therefore, we are actually comparing our method with a widely recognized template.

Since the differences in anxiety relief levels were not significant in all groups involved in the analyses, the participant satisfaction in this experiment might be decoupled from the observed level of anxiety relief. This finding suggests a potential dissociation between user satisfaction and the actual intervention efficiency. However, from the perspective of feasibility, this kind of dissociation might provide a preliminary signal of personalization for specific user groups. By pointing out the limitations in user preference of model recommendation, the identification of target user group in the future might benefit from this finding.

Although universal advantages were not observed across all subgroups, the model stability analysis (‘Model Stability Analysis’) suggested that the model captured relevant patterns in the training data. The technical robustness shows that the personalization framework for deep breathing intervention is viable and reproducible, therefore it may serve as a foundation for personalized interaction designs. Future studies with larger and more diverse samples, bigger and more advanced model implementations, as well as more sensitive outcome measures, is necessary for conducting a better evaluation and implementation of personalized interventions built on this robust modeling approach.

### Limitations

**Figure 5:**
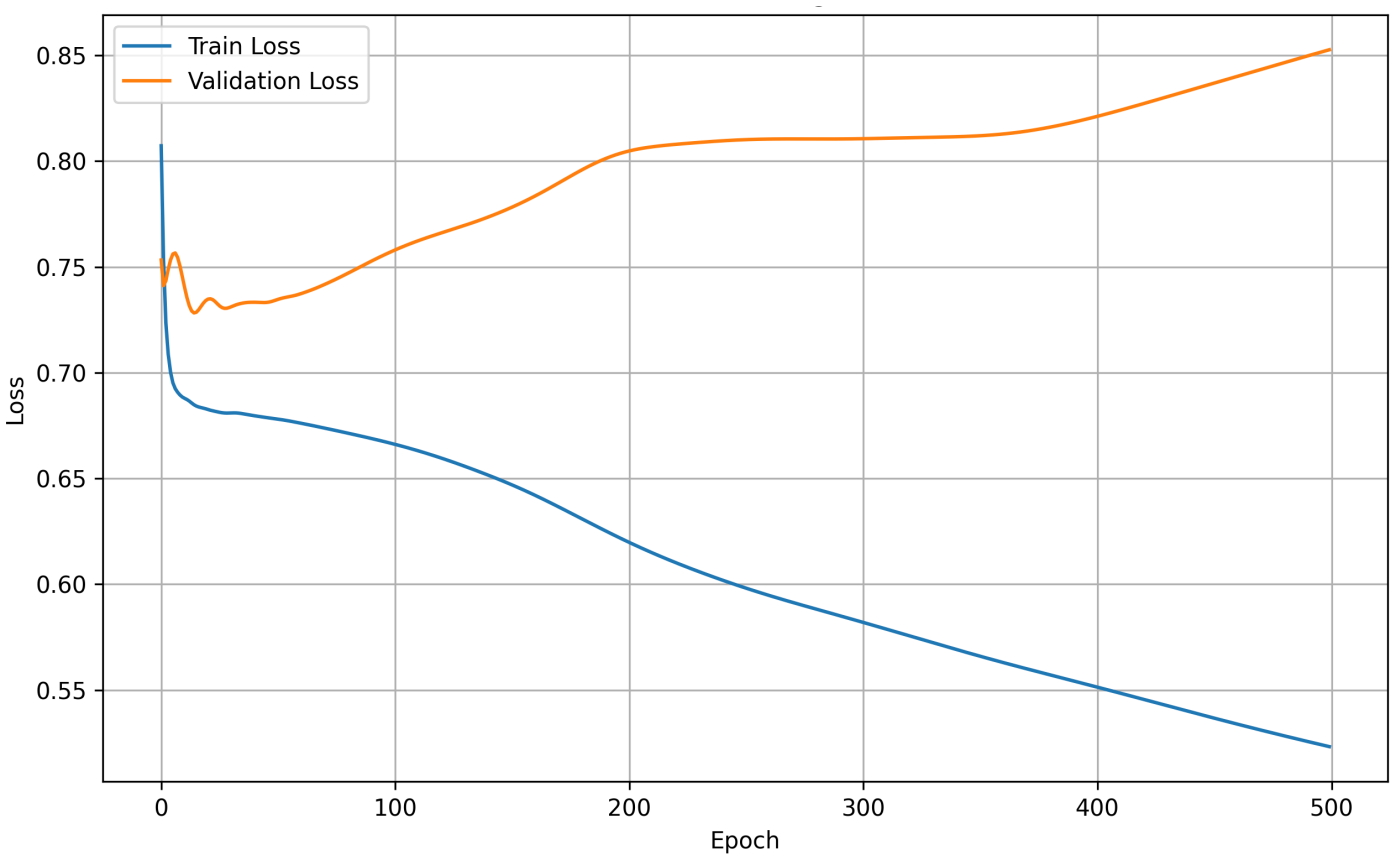
Training and evaluation loss curve of the model.

This study has several notable limitations. First, during the training of the model, we noticed that, as Fig. 5 shown, even though the training loss was decreasing stably, the evaluation loss diverged, which informs us of the problem of overfitting. As the study of Hawkins (2004) has shown, overfitting might lead to worsening the model prediction results.

In the retrospective review of the system deployment, we noticed that there is a minor implementation oversight regarding the sign of a term in Eq. (1). The platform used for data collection has since been taken offline as the original experiment has concluded. While the implementation error and overfitting issue were corrected for the stability analysis in ‘Model Stability Analysis’, they remain a noted limitation of the evaluation phase. Nevertheless, our comparative results suggest that the core logic of the model remains robust despite these technical nuances, though further refinement in future large-scale evaluations is warranted.

Additionally, the parameters *λ*_1_, *λ*_2_,  and *λ*_3_ of the loss function referred to in ‘Design of Custom Loss’ are decided based on our experience and expectation, which might not be the optimal.

For training of the model, we deliberately filtered the collected data for more positive outcome (see ‘Data Pre-processing’), which introduced selection bias to the model. As the study of Hernán, Hernández-Díaz & Robins (2004) discovered, selection bias might lead to a mismatch between the collection participants and the real users. Also, we approximated the intervention duration of the fixed template group to the mean duration of the model’s recommendations, which might decrease the difference between these approaches.

Second, the evaluation only relied on the self-reported experiences of the participants and did not include objective physiological indicators. Even though we added attention checks to the questionnaires, they could sometimes still be randomly reported. Physiological indicators are thus necessary for a better measurement of anxiety level and could not be completely replaced by questionnaires, if specialized hardware is available. Regarding this problem, we also recognized that our data pre-processing procedure was minimal and did not filter all of the invalid or inattentive responses, which could compromise the data quality. However, we didn’t collect enough samples for every subgroup. As a result, some subgroups had insufficient sample sizes, which limited the reliability and robustness of statistical analyses. In addition, more information about participants (*e.g.*, medication use or caffeine intake) should also be collected for better measuring the effectiveness.

Last, our research scope was explicitly restricted to the feasibility of data-driven immediate non-medical intervention for anxiety relief. Therefore, the longitudinal efficacy and sustained benefits of applying the model-recommended intervention remain unexplored. Since our study only focuses on non-medical intervention, the medical efficiency of our approach remains unexplored.

### Future research

Future studies can improve the reliability and applicability of the model by addressing the aforementioned limitations through a more rigorous model selection and loss function design, improving the data collection protocols, enhancing the data cleaning procedures, integrating biological indicators with participant evaluations, expanding the sample, and appropriately training the model.

Additionally, as the results of sensitivity analysis of GAD-2 in Appendix A suggested, incorporating trait anxiety as a factor may enhance the efficacy of this approach in future studies.

## Conclusion

This study explored the feasibility of data-driven breathing intervention recommendations for anxiety relief with a hardware-free, easily accessible approach. This method was compared to the proven effectiveness of a conventional fixed template baseline.

Methodologically, this study represents an effort to integrate machine learning with personalized breathing intervention. By independently deploying a full-stack platform to conduct the intervention, we maintained rigorous experimental control when comparing fixed templates with model recommendations. To our knowledge, this establishes a foundational framework for adaptive, personalized intervention strategies specifically targeting the reduction of anxiety. These methodological advances lay the groundwork for future studies aiming to further optimize and personalize digital mental health interventions.

The model stability analysis confirmed consistent and reliable outputs across different model initializations. For different models trained using the same method, there is strong agreement between the results they generate (*mean* *CV* < 10%). However, this methodological robustness should not be interpreted as evidence of improved intervention effectiveness. The evaluation demonstrated no statistical significant difference in effectiveness and participant satisfaction between model recommendation and fixed templates. Furthermore, subgroup analyses revealed variations based on participant characteristics.

Our proposed method demonstrated stable performance on the available dataset and may serve as a reasonable approach to implementing personalized recommendations. However, our approach did not show a universal superiority. This may be attributed to factors such as model overfitting, selection bias of training data, or lack of precise data filtering. Future research can focus on enhancing the diversity and quality of training data, trying advanced Machine Learning or offline Reinforcement Learning approaches, integrating physiological metrics, and refining personalization techniques to maximize the intervention effectiveness across broader populations.

##  Supplemental Information

10.7717/peerj.21483/supp-1 Supplemental Information 1 Raw data of the evaluation experiment

10.7717/peerj.21483/supp-2 Supplemental Information 2 Codebook for evaluation experiment data

## Appendix A. Sensitivity analysis of GAD-2

Although as ‘Data Collection for Training’ referred to, we omitted the GAD-2 data from training data collection questionnaire and removed this scale in evaluation data collection questionnaire, this sensitivity analysis is conducted, in order to explore the potential influence of long-term trait anxiety level on our breathing intervention method.

### Model stability analysis

Consistent with the approach in ‘Model Stability Analysis’, we conducted an inter-model variance across 84,840 synthetic test samples. The test results are shown in Table 5. The CV values for the inhalation, exhalation, and repetition outputs are all below 10%, which suggests a high measurement consistency across models with GAD-2 scores as additional feature.

### Output difference analysis

We used the same approach as ‘Exploratory Subgroup Analysis’, *i.e.,* Brunner-Munzel test with FDR correction as well as CI-calculation by Bootstrapping, to analyze the statistical difference in the output variables Inhalation, Exhalation, and Repetition. The test result is shown in Table 6.

The test result showed that even though all the differences are statistically significant, the difference in the output variable Inhalation is negligible (|Cliff’s delta| < 0.147). Additionally, there is a small effect size in Exhalation (|Cliff’s delta| < 0.33) and a medium effect size in Repetition (|Cliff’s delta| ≥ 0.33). This test result shows that with GAD-2 score as an additional input feature, the model adaptively recommends breathing interventions with longer exhalation time and more repetition. This finding suggests that taking long-term trait anxiety into account might affect the output of the model, and whether these differences improve intervention effectiveness requires further evaluation.

**Table 5:** Mean CV values for each primary output across models with GAD-2 scores as additional feature. Lower CV values indicate higher model output stability.

Variable	Mean CV (%)
Inhalation	4.95
Exhalation	5.44
Repetition	9.53

**Table 6:** Differences in all measured variables between models with and without GAD-2 score as input feature, using synthetic input data as referred in Model ‘Stability Analysis’ and ‘Model Stability Analysis’.

Variable	*P*-value	Cliff’s Delta [95% CI]
Inhalation	2.6 × 10^−67^	−0.077 [−0.086, −0.069]
Exhalation	<0.001	0.293 [0.285, 0.302]
Repetition	<0.001	0.515 [0.505, 0.523]

## Additional Information and Declarations

### Data Availability

The following information was supplied regarding data availability: The raw data is available in the Supplemental File. The code is available at Zenodo: - gbzdy0416. (2026). gbzdy0416/ml4breath: 1.0 (Initial). Zenodo. https://doi.org/10.5281/zenodo.20047426
- gbzdy0416. (2026). gbzdy0416/data_processing4ml4breathing: 1.0 (initial). Zenodo. https://doi.org/10.5281/zenodo.20047409.
