# Supplementary material for: Personalized breathing recommendations for anxiety relief: a data-driven exploratory approach focusing on feasibility
Source: PeerJ. 2026 Aug 4;14:e21483. doi: 10.7717/peerj.21483 (PMC13450033; doi:10.7717/peerj.21483)
Supplement: Supplementary file 1 [file peerj-14-21483-s002.pdf]

does not apply to me; does rather not apply to me; does partly apply to me; does totally apply to me

row2_responseCode (STAI scale)	
1	not at all
2	a little
3	somewhat
4	very much so
row5_responseCode (Attention Check)	
1	Spade (valid data point)
2	Heart (invalid data point)
3	Diamond (invalid data point)
4	Club (valid data point)
row7_responseCode (CSQ-4 Scale)	
1	does not apply to me
2	does rather not apply to me
3	does partly apply to me
4	does totally apply to me
row8_responseCode (Age Group)	
1	18-24
2	25-30
3	31-40
4	41-50
5	51-60
6	60+
7	Prefer not to say
row9_responseCode	
1	Male
2	Female
3	Divers
4	Other
5	Prefer not to say
